# Total body water to lean body mass ratio predicts mortality in patients with chronic heart failure: a prospective, observational study

**DOI:** 10.3389/fnut.2026.1762912

**Published:** 2026-03-04

**Authors:** Linfeng Xie, Bryan Richard Sasmita, Yuhe Zhao, Jing Chen, Yuanzhu Li, Suxin Luo, Bi Huang

**Affiliations:** 1 Department of Cardiology, The First Affiliated Hospital of Chongqing Medical University, Chongqing, China; 2 Department of Cardiology, Chongqing Seventh People’s Hospital, Chongqing, China

**Keywords:** all-cause mortality, cardiovascular mortality, chronic heart failure, lean body mass, total body water

## Abstract

**Background:**

Malnutrition and sodium water retention are some of the most common complications of chronic heart failure (CHF). To date, several parameters or risk stratification tools have been established to predict one’s volume or nutritional status. Unfortunately, there is no biomarker that may reflect both conditions, thus, in this study, we established a novel biomarker known as total body water (TBW) to lean body mass ratio (LBM) ratio (TLR). Accordingly, we also assessed the prognostic value of TLR in CHF patients.

**Methods:**

A total of 401 consecutive patients with CHF from August 2019 to October 2021 were prospectively enrolled. TBW and LBM were obtained by InBody S10. The primary endpoint was long-term all-cause and cardiovascular mortality. The cut-off and prognostic value of TLR was determined by receiver operating characteristic curves and Cox regression analysis. Patients were then divided into two groups according to the cut-off value of TLR.

**Results:**

During a median follow-up of 1,200 days, the high-TLR group (TLR ≥ 0.783) was presented with a higher all-cause mortality (41.27% vs. 18.40%, *p* < 0.001) and cardiovascular mortality (28.57% vs. 13.68%, *p* < 0.001) compared to the low-TLR group (TLR < 0.783). Furthermore, patients in the high-TLR group tended to be older, presented with atrial fibrillation, had higher NYHA class, had a history of chronic kidney disease, had a higher level of N-terminal prohormone of brain natriuretic peptide, worse nutritional status, and a lower level of albumin (all *p* < 0.05). The Kaplan–Meier curves of the two group patients revealed that the cumulative all-cause and cardiovascular mortality were lower in patients with lower TLR (all log-rank *p* < 0.001). In the multivariate Cox proportional hazard analysis, TLR ≥ 0.783 was an independent predictor for both all-cause mortality (HR = 2.108, 95%CI 1.400, 3.173, *p* < 0.001) and cardiovascular mortality (HR = 2.044, 95%CI 1.264, 3.305, *p* = 0.004).

**Conclusion:**

The TLR may serve as a novel composite biomarker that reflects both volume and nutritional status in CHF patients and is associated with long-term prognosis. Its prognostic performance appears comparable to several established biomarkers, though further validation is warranted.

## 1 Introduction

Congestive Heart Failure (CHF) is a clinical syndrome characterized by dysfunction of the left ventricle, resulting in fluid retention and inadequate perfusion of vital organs. Fluid congestion is an important pathophysiologic mechanism in CHF, as it leads to cardinal symptoms such as dyspnea, pulmonary congestion, pitting edema, and elevated jugular venous pressure (1). The presence of such symptoms not only impairs patients’ quality of life but is also associated with higher risks of re-hospitalization and worse outcomes (2, 3). Cardiac catheterization is the golden standard to assess one’s volume status. However, invasiveness, cost, and accessibility limit their clinical use (4). Echocardiography and natriuretic peptides have been proven to be able to evaluate the volume status of CHF patients (5), however, such parameters only give a rough estimation of body fluid content and are often insensitive.

Persistent insufficient cardiac output may cause reduced forward blood flow and impaired vasodilation, eventually leading to inadequate skeletal muscle and vital organ perfusion, which is a hallmark of tissue ischemia. Such a chronic state of malperfusion is prone to cause muscle wasting with or without affecting fat tissue, known as cardiac cachexia. Cardiac cachexia is a type of malnutrition and muscle loss that affects about 10–39% of people with CHF (6). The annual mortality of cardiac cachexia is about 20–40% (7), with a study found an increase in mortality rate by 50% within 18 months of first diagnosis (8), making it one of the leading causes of mortality in CHF patients. Several serological biomarkers, as well as nutritional risk stratification scores to aid early detection and improve the prognosis of cardiac cachexia have been identified (9–14). Unfortunately, such complex algorithms and unspecific biomarkers hinder their application in the clinical setting or bedside use. Hence, more accurate and specific biomarkers to reflect one’s volume and nutritional status are needed to improve the prognosis of such patients.

Body impedance analysis (BIA) is a simple, safe, and non-invasive test for estimating body composition, in particular muscle mass, and volume status (15). BIA measures total body resistance at different frequencies and estimate total body water, lean body mass, extracellular fluid, fat mass, and other body compartments (16, 17). Lean body mass (LBM) represents non-adipose tissue and mainly composed of total body water (TBW) and muscle mass (18). These two components provided a significant meaning in the pathophysiology of HF. Previous studies have found that lower LBM was associated with a higher long-term mortality in CHF patients (19), while, other studies focused on the prognostic value of extracellular water to TBW ratio (20, 21). In this study, we proposed a new biomarker, which is TBW to LBM ratio (TLR). The fundamental of this parameter purely based on the pathophysiology of CHF-induced fluid congestion and malnutrition. We believe that TLR not only able to reflect the hydration level and nutritional status of CHF patients, but may also serve as a new prognostic biomarker.

## 2 Methods

### 2.1 Study design and population

This study was a single-center, prospective observational study with the aim to evaluate the usefulness of TLR for evaluating the prognosis in patients with CHF. The study protocol was approved by the Ethics Committee of the First Affiliated Hospital of Chongqing Medical University and confirmed to the Declaration of Helsinki. All patients have provided written informed consent.

From August 2019 to October 2021, 401 consecutive patients were admitted and enrolled in this study due to CHF. The eligible patients were men and women aged 18 years and older who presented with stable symptomatic CHF of four or more weeks’ duration. The main exclusion criteria were acute HF, septic shock, hypovolemic shock, congenital heart disease, and anaphylactic shock.

### 2.2 Data collection

After admission, echocardiography was performed within 24 h, and blood samples were collected immediately after admission and were tested in the central laboratory. Then, baseline characteristics, the results of echocardiography and blood tests were collected by experienced clinicians from the computerized patient record system.

### 2.3 Multifrequency BIA

The BIA was performed within 8 h after admission using segmental and multi-frequency InBody S10 (InBody China, Beijing, China). Eight electrodes were affixed to the patient’s body: four were placed on the bases of the middle fingers and the dorsal surfaces of the wrists, and the remaining four were placed on the bases of the middle toes and the anterior surfaces of the ankles (22). Thirty impedance measurements were made by analyzing the conductance of the electrical current across five body segments (legs, arms, and trunk) at multiple frequencies (1, 5, 50, 250, 500, and 1,000 kHz). High frequencies flow through cell membranes and along the entire body of water, while low frequencies primarily flow via extracellular water, allowing detection of the water content outside of the cells. Total and regional body compositions including LBM, fat body mass, bone mineral content, and other body compartments were also assessed using InBody S10. TLR was defined as a ratio between total body water to lean body mass that were measured using InBody S10.

### 2.4 Study endpoints

The primary endpoint of this study was long-term all-cause and cardiovascular mortality.

### 2.5 Statistical analysis

Based on the distribution of data, continuous variables presented in mean value and standard deviations or median value and inter-quartile range (25th and 75th) as appropriate, meanwhile, the inter-group comparison was conducted by two independent sample *t*-test or Mann–Whitney *U* test. Categorical variables were presented in numbers and percentages, and Chi-Square test or Fisher test was employed for inter-group comparison. Boruta feature selection assessed the relationship between TLR, and clinically significant covariates with respect to the primary outcome. The area under the curve (AUC) of all-cause and cardiovascular mortality was calculated by the receiver operating characteristic curve (ROC) to determine the cut-off value of TLR. Thereafter, patients were divided into two groups according to the cut-off value of TLR. To assess the prognostic value of TLR, the survival analysis of Kaplan–Meier (K–M) curves were constructed, followed by the log-rank test. Logistic and linear regression analyses were used to analyze the association between LBM with body water, as well as the association between TLR with previously confirmed biomarkers [e.g., age, left ventricular ejection fraction (LVEF), cTnI, N-terminal prohormone of brain natriuretic (NT-proBNP), etc.] (5, 23). Furthermore, to compare the prognostic performance of TLR with established individual indicators, including hemoglobin, albumin, creatinine, body mass index, NT-proBNP, NYHA class, LVEF, controlling nutritional status (CONUT) score, prognostic nutritional index (PNI) and the geriatric nutritional risk index (GNRI), we performed ROC curve analyses and compared the AUC. To quantify the improvement in discrimination when using TLR versus each conventional marker, we calculated the Integrated Discrimination Improvement (IDI). IDI was selected because it directly evaluates the difference in discriminative ability between two independent models without relying on predefined risk thresholds or a common baseline model, making it suitable for comparing single biomarkers.

The restricted cubic spline analysis (RCS) for the association between TLR and primary endpoint was performed. Univariate and multivariate Cox regression models were constructed to investigate whether there was an independent relationship between TLR and primary endpoints. The multivariate Cox regression model included age, gender, NYHA class, atrial fibrillation, NT-proBNP, LVEF, moderate–severe mitral regurgitation, ICW, ECW, and the use of diuretics. Subgroup analyses were conducted to assess the impact of ELR on all-cause mortality in subgroups patients based on age, gender, NYHA class, hypertension, diabetes, renal dysfunction, HF types, HF etiologies, the use of diuretic, BMI, COUNT score, GNRI, and PNI. To assess the robustness of the primary TLR cut-off (≥0.783) and its association with mortality, we performed sensitivity analyses using alternative classifications of TLR. Specifically, we categorized patients into tertiles based on TLR distribution and repeated the Cox regression analyses using the lowest tertile as the reference group. These analyses aimed to verify that the observed relationship between elevated TLR and poor prognosis was not solely dependent on the chosen ROC-derived threshold. All statistical analyses were carried out using the SPSS statistical software, version 25.0 (IBM, Armonk, NY, United States), MedCalc statistical software 19.2.6 (Ostend, Belgium), GraphPad Prism 8.4.3 (GraphPad Software, Inc., San Diego, CA, United States), and R (version 3.6.3, R foundation for statistical computing, Vienna, Austria).

## 3 Results

From August 2019 to October 2021, a total of 409 consecutive patients were diagnosed with CHF, among which eight patients had incomplete data or loss of follow-up, and the remaining 401 patients with CHF were included in this study. During the 1,200-day follow-up period, the all-cause mortality and cardiovascular mortality were 29.18% (117 patients) and 20.70 (83 patients).

**Figure 1:**
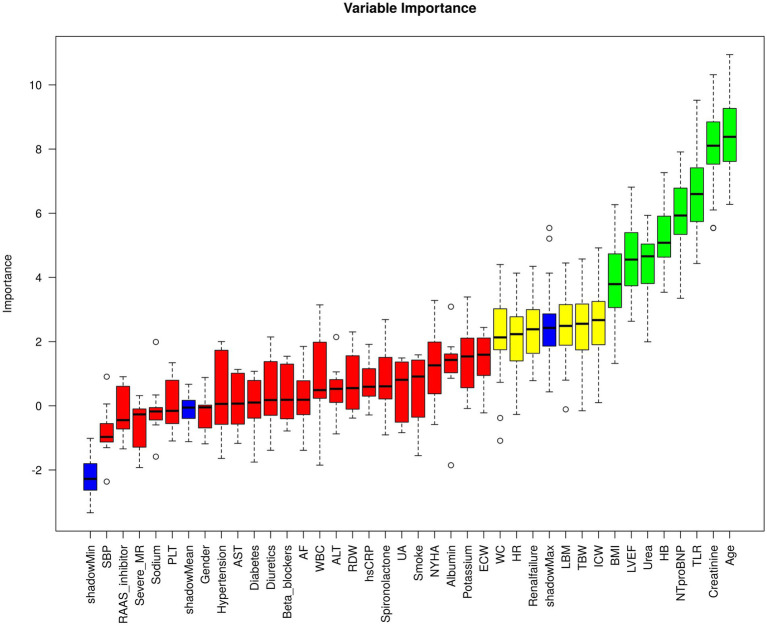
Boruta feature selection between clinically relevant covariates with the primary outcome. The plot shows the feature importance rankings after applying the Boruta algorithm to the selected variables. Each feature’s importance is represented by a bar, with green bars indicating important features, red bars indicating unimportant features, and yellow bars representing uncertain features. LVEF, left ventricular ejection fraction; NT-proBNP, N-terminal prohormone of brain natriuretic peptide; BMI, body mass index; ELR, extracellular water to lean body mass ratio; HB, hemoglobin; LBM, lean body mass; MR, mitral regurgitation; WBC, white blood cell; WC, waist circumference; AST, aspartate aminotransferase; HR, heart rate; ALT, alanine aminotransferase; SBP, systolic blood pressure; AF, atrial fibrillation.

**Figure 2:**
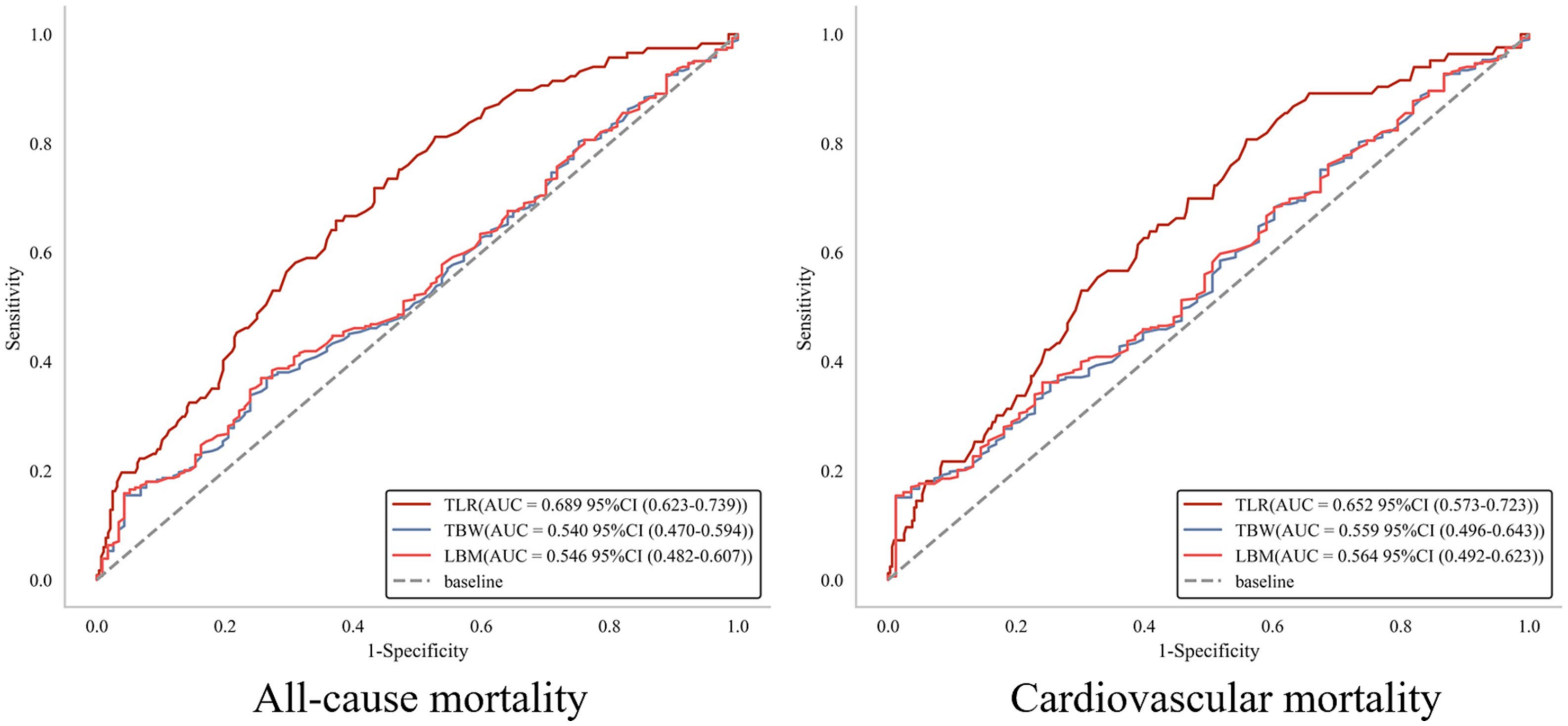
ROC analysis of total body water (TBW), lean body mass (LBM), and total body water to lean body mass ratio (TLR) for predicting long-term all-cause and cardiovascular mortality.

Boruta analysis identified TLR as a key predictor of all-cause mortality in CHF patients (Figure 1). The present study aimed to evaluate the predictive value of TLR in CHF patients; therefore, ROC analysis was performed. As shown in Figure 2, the TLR (AUC for all-cause mortality: 0.689, for cardiovascular mortality: 0.652) was presented with a modest and higher predictive value compared to LBM (AUC for all-cause mortality: 0.546, for cardiovascular mortality: 0.564) and TBW (AUC for all-cause mortality: 0.540, for cardiovascular mortality: 0.559). In head-to-head comparisons, TLR showed discriminative performance that was comparable to or modestly better than several conventional prognostic markers in our cohort, though its absolute discriminative ability remained moderate. The AUC for TLR was higher than other prognostic biomarkers (Supplementary Figure S1). The IDI analysis further demonstrated that TLR provided statistically significant improvement in discrimination compared to most conventional indicators, however, the magnitude of improvement was modest and its advantage over certain markers such as NT-proBNP and NYHA class was less pronounced (Supplementary Table S2).

The optimal cut-off value of TLR ≥ 0.783 was determined by ROC analysis and Youden index to maximize sensitivity (65.8%) and specificity (62.7%) for mortality prediction in our cohort. We recognize that this threshold may be influenced by the specific characteristics of our study population and the use of the InBody S10 device for body composition assessment. Based on this cut-off, the population was stratified into low TLR (<0.783) and high TLR (≥0.783) groups.

The baseline characteristics, the results of echocardiography and blood tests were compared (Table 1). The patients in the high TLR group tended to be older, presented with atrial fibrillation, and had higher NYHA class (all *p* < 0.05). As for medical histories, more patients in the high TLR group had a history of chronic kidney disease (*p* = 0.001), but less patients had a history of dyslipidemia (*p* = 0.002) and dilated cardiomyopathy (*p* = 0.026). However, there was no significant difference for hypertension, diabetes mellitus, previous myocardial infarction, previous percutaneous coronary intervention (PCI), previous coronary artery bypass grafting (CABG), and chronic obstructive pulmonary disease (COPD) (all *p* > 0.05). Moreover, the results of echocardiography showed that patients with high TLR had larger right atrium, right ventricle, and left atrium (all *p* < 0.05), and a higher percentage of patients presented with moderate to severe mitral regurgitation in them (*p* = 0.010). In terms of laboratory parameters, patients in the high TLR group had a higher level of NT-proBNP, D-dimer, urea, creatinine, and uric acid (all *p* < 0.05), while the level of white blood cell, hemoglobin, albumin, and platelet was lower (all *p* < 0.05). Body compartment analysis measured by BIA was presented in Table 2. TBW, mineral, LBM, and body fat were comparable between the two groups, while waist circumference, ICW, ECW, protein, and edema index were statistically significant (all *p* < 0.05). Furthermore, patients in high TLR group had worse nutritional status as demonstrated by CONUT score, PNI, and GNRI (Supplementary Table S1).

**Table 1:** A comparison of baseline characteristics of the 2 groups.

Baseline characteristics	Total *n* = 401	TLR < 0.783 *n* = 212	TLR ≥ 0.783 *n* = 189	*p*-value
Age (years)	69.51 ± 13.74	66.06 ± 14.01	73.37 ± 12.37	<0.001
Male (%)	250 (62.3)	138 (65.1)	112 (59.3)	0.323
BMI (kg/m^2^)	23.97 ± 4.09	24.19 ± 3.68	23.72 ± 4.50	0.253
Smoking (%)	174 (43.4)	94 (44.3)	80 (42.3)	0.685
Alcohol drinking (%)	134 (33.4)	75 (35.4)	59 (31.2)	0.378
Family History of CVD (%)	52 (13.0)	30 (14.2)	22 (11.6)	0.455
Atrial Fibrillation (%)	160 (39.9)	67 (31.6)	93 (49.2)	<0.001
NYHA Class (%)				<0.001
1	29 (7.2)	24 (11.3)	5 (2.6)	
2	94 (23.4)	60 (28.3)	34 (18.0)	
3	185 (46.1)	90 (42.5)	95 (50.3)	
4	93 (23.2)	38 (17.9)	55 (29.1)	
Ischemic Heart Disease (%)	171 (42.7)	92 (43.4)	79 (41.8)	0.747
Dilated Cardiomyopathy (%)	64 (16.0)	42 (19.8)	22 (11.6)	0.026
Medical histories (%)				
Hypertension (%)	206 (51.4)	105 (49.5)	101 (53.4)	0.434
Diabetes mellitus (%)	131 (32.7)	62 (29.3)	69 (36.5)	0.301
COPD (%)	29 (7.2)	13 (6.1)	16 (8.5)	0.368
Dyslipidemia (%)	39 (9.7)	30 (14.2)	9 (4.8)	0.002
Previous MI (%)	46 (11.5)	29 (13.7)	17 (9.0)	0.142
Previous PCI (%)	62 (15.5)	29 (13.7)	33 (17.5)	0.296
Previous CABG (%)	3 (0.7)	2 (0.9)	1 (0.5)	1.000
Chronic Kidney Disease (%)	104 (25.9)	40 (18.9)	64 (33.9)	0.001
Laboratory parameters				
Cardiac Troponin I (ng/mL)	0.05 (0.01–0.79)	0.05 (0.02–1.63)	0.05 (0.01–0.21)	0.190
NT-proBNP (pg/mL)	1,500 (599–4,045)	1,180 (468–3,190)	2035 (1013–4,783)	<0.001
D-dimer (ng/mL)	685 (233–1,316)	534 (219–1,095)	856 (322–1,625)	0.004
Leukocyte (×10^9^/L)	6.56 (5.40–8.76)	6.78 (5.60–9.07)	6.34 (4.99–8.30)	0.006
Hemoglobin (g/L)	130 (113–145)	136 (123–148)	124 (105–141)	<0.001
Platelet (×10^9^/L)	172 (141–222)	179 (150–230)	165 (127–213)	0.003
Albumin (g/L)	39 (36–42)	40 (37–43)	37 (34–40)	<0.001
Urea (mmol/L)	8.1 (6.2–12.0)	7.2 (5.9–10.0)	9.5 (6.8–13.7)	<0.001
Creatinine (μmol/L)	93 (73–31)	87 (71–112)	105 (80–154)	<0.001
Uric Acid (μmol/L)	428 (336–562)	404 (320–518)	458 (354–595)	0.001
Echocardiography				
Right Atrium (mm)	40.86 ± 6.98	39.39 ± 6.31	42.51 ± 7.34	<0.001
Right Ventricle (mm)	22.43 ± 4.12	21.76 ± 3.44	23.19 ± 4.67	0.001
Left Atrium (mm)	41.06 ± 8.42	39.74 ± 7.54	42.54 ± 9.11	0.001
LVEDD (mm)	57.34 ± 10.27	57.79 ± 10.07	56.84 ± 10.49	0.352
IVS (mm)	10.58 ± 2.78	10.51 ± 2.46	10.66 ± 3.17	0.598
Moderate to severe MR (%)	183 (45.6)	84 (39.6)	99 (52.4)	0.010
EF (%)	43.70 ± 12.87	42.79 ± 12.24	44.73 ± 13.50	0.133

TLR, total body water to lean body mass ratio; BMI, body mass index; CVD, cardiovascular diseases; NYHA, New York Heart association Functional Classification; COPD, chronic obstructive pulmonary disease; MI, myocardial infarction; PCI, percutaneous coronary intervention; CABG, coronary artery bypass graft surgery; NT-proBNP, N-terminal prohormone of brain natriuretic peptide; LVEDD, left ventricular end-diastolic diameter; IVS, intraventricular septum; MR, mitral regurgitation; FS, fractional shortening; EF, ejection fraction.

**Table 2:** A comparison of bioelectrical impedance analysis of the 2 groups.

Characteristics	Total *n* = 401	TLR < 0.783 *n* = 212	TLR ≥ 0.783 *n* = 189	*p*-value
Height (cm)	160.96 ± 8.34	161.13 ± 7.88	160.77 ± 8.85	0.664
Weight (kg)	62.22 ± 13.05	63.17 ± 12.27	61.15 ± 13.82	0.123
Waist Circumference (cm)	77.12 ± 12.89	79.64 ± 12.72	74.30 ± 12.51	<0.001
Intracellular water (kg)	20.55 ± 4.86	21.21 ± 4.99	19.81 ± 4.61	0.004
Extracellular water (kg)	13.45 ± 3.13	13.06 ± 2.86	13.88 ± 3.36	0.009
Total body water (kg)	34.00 ± 7.81	34.27 ± 7.72	33.69 ± 7.91	0.453
Protein (kg)	8.89 ± 2.10	9.12 ± 2.15	8.56 ± 2.00	0.003
Mineral (kg)	2.83 ± 0.70	2.83 ± 0.74	2.83 ± 0.65	0.951
Lean body mass (kg)	43.44 ± 10.01	44.01 ± 9.98	42.79 ± 10.03	0.222
Edema index (%)	39.60 ± 2.1	38.2 ± 1.8	41.2 ± 1.3	<0.001
Body fat mass (kg)	15.95 ± 8.48	16.33 ± 7.91	15.53 ± 9.09	0.346
Body fat percentage (%)	25.06 ± 11.29	25.51 ± 10.66	24.56 ± 12.00	0.403

TLR, total body water to lean body mass ratio.

**Figure 3:**
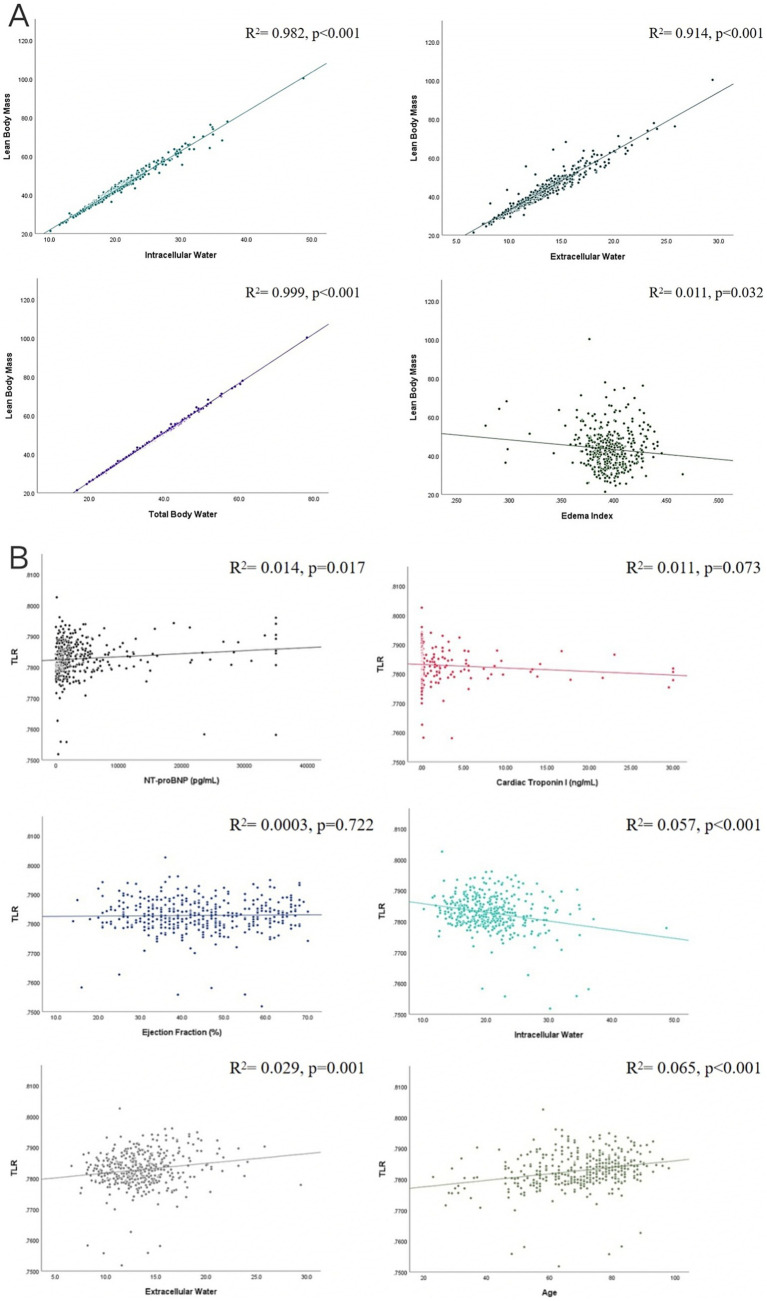
Correlation analysis. **(A)** Correlations between lean body mass (LBM) and intracellular water (ICW), extracellular water (ECW), total body water (TBW), and edema index. **(B)** Correlation analysis between total body water to lean body mass (TLR) and left ventricular ejection fraction, cardiac troponin I, intracellular water (ICW), age, NT-proBNP, and extracellular water (ECW).

Correlations between LBM and body fluid content were presented in the Figure 3A. It showed that LBM was positively associated with ICW (*R*^2^ = 0.982, *p* < 0.001), ECW (*R*^2^ = 0.914, *p* < 0.001), and TBW (*R*^2^ = 0.999, *p* < 0.001), however, LBM was negatively associated with edema index (*R*^2^ = 0.011, *p* = 0.032). Figure 3B displayed the correlation analysis between TLR with other CHF biomarkers. We found that TLR had no correlation with LVEF (*R*^2^ = 0.0003, *p* = 0.722) and cardiac troponin I (*R*^2^ = 0.011, *p* = 0.073), negatively associated with ICW (*R*^2^ = 0.057, *p* < 0.001), and positively associated with age (*R*^2^ = 0.065, *p* < 0.001), NT-proBNP (*R*^2^ = 0.014, *p* = 0.017), and ECW (*R*^2^ = 0.029, *p* = 0.001).

**Figure 4:**
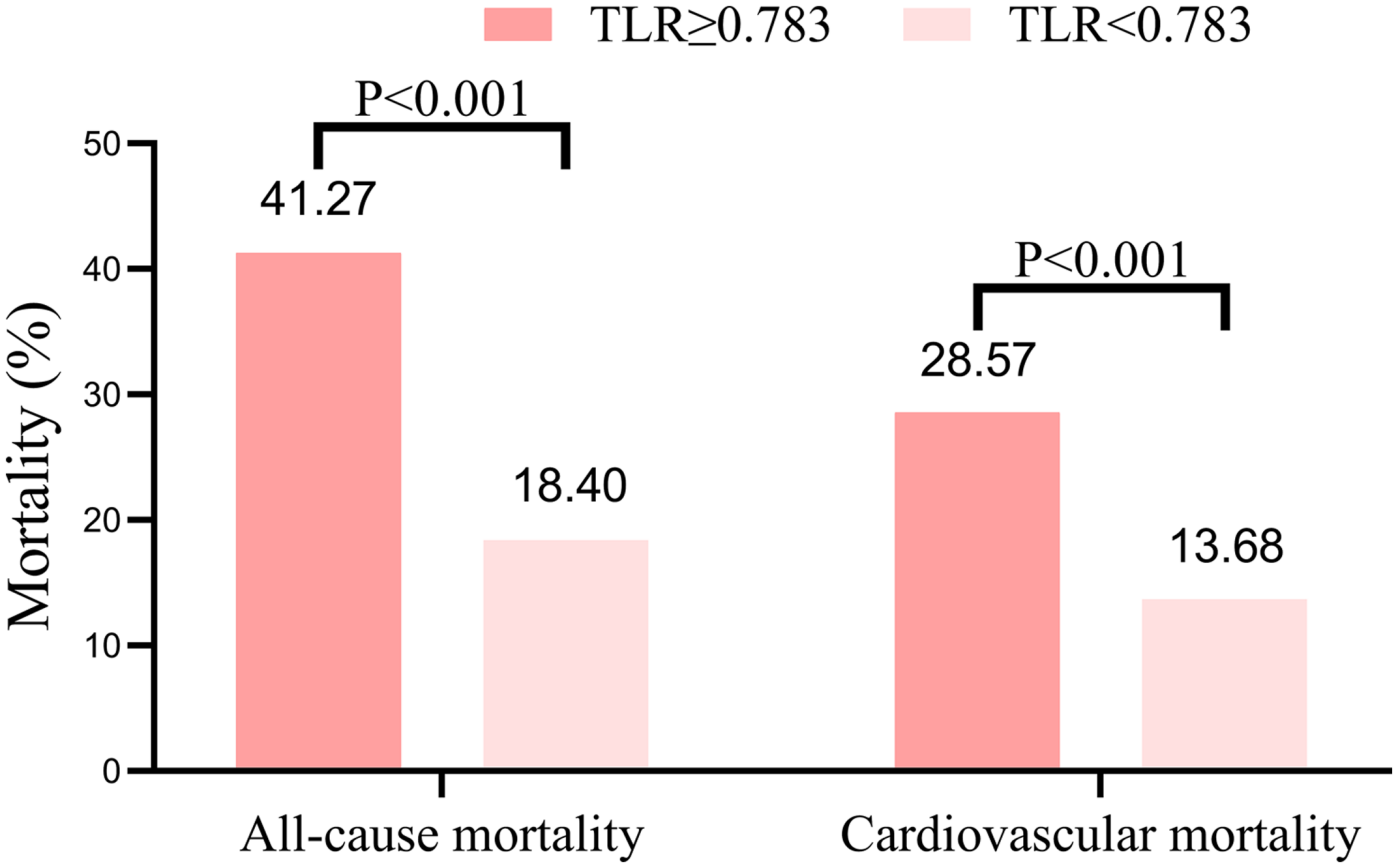
Long-term all-cause and cardiovascular mortality according to the total body water to lean body mass ratio (TLR).

**Figure 5:**
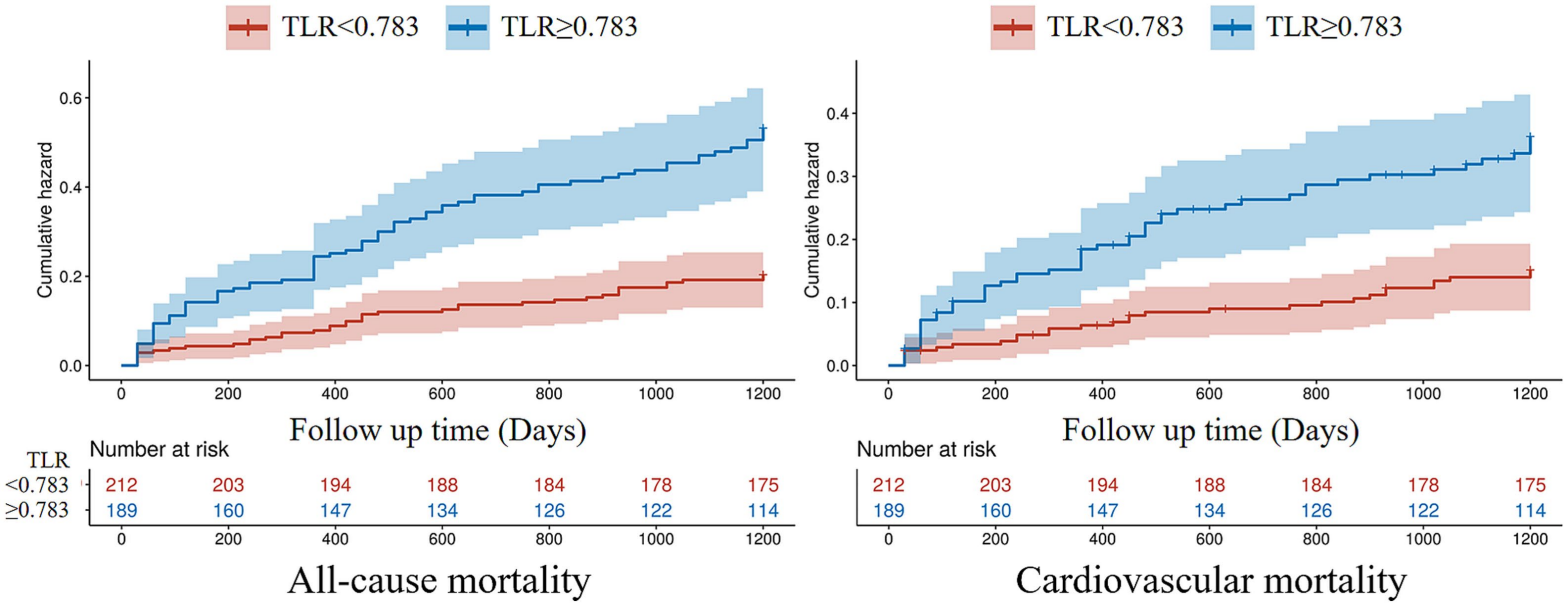
Kaplan–Meier curves for all-cause and cardiovascular mortality. TLR: total body water to lean body mass ratio.

**Figure 6:**
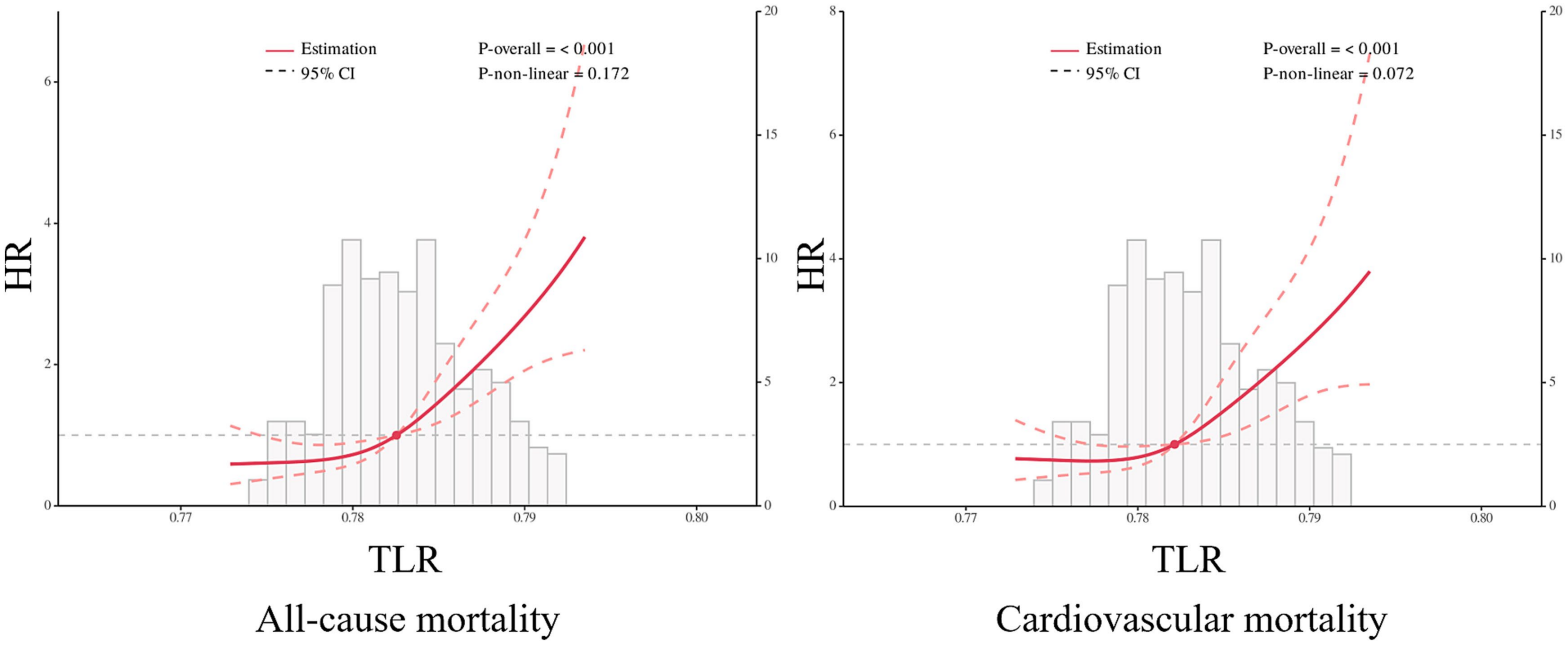
Potential nonlinear for the levels of total body water to lean body mass ratio (TLR) with all-cause and cardiovascular mortality measured by restricted cubic spline.

During the 1,200-day follow-up period, the all-cause and cardiovascular mortality rate was significantly higher in the high TLR group compared to the low TLR group (all-cause mortality rate 41.27% vs. 18.40%, *p* < 0.001, and cardiovascular mortality rate 28.57% vs. 13.68%, *p* < 0.001, respectively, Figure 4). The K–M curves of the two group patients revealed the cumulative all-cause and cardiovascular mortality were lower in patients with lower TLR (all log-rank *p* < 0.001, Figure 5). The results of the RCS for the association of TLR with the primary endpoint were presented in Figure 6. A higher level of TLR was linearly associated with a higher risk of all-cause and cardiovascular mortality.

Univariate and multivariate Cox regression models were constructed to confirm the relationship between TLR and study endpoints (Tables 3, 4). As for all-cause mortality, the univariate Cox proportional hazard analysis showed that age (>65 years old), body mass index (BMI), NYHA class ≥ 3, ICW, and TLR (≥0.783) were associated all-cause mortality (all *p* < 0.05, Table 3). After multivariate adjustment, TLR ≥ 0.783 was still an independent prognostic factor for all-cause mortality (HR = 2.108, 95%CI 1.400, 3.173, *p* < 0.001, Table 3). The other independent predictors included age, BMI, NYHA class ≥ 3 (Table 3). Furthermore, the univariate Cox proportional hazard analysis showed that BMI, NYHA ≥ 3, moderate–severe mitral regurgitation, ICW, and TLR ≥ 0.783 were associated with cardiovascular mortality (all *p* < 0.05, Table 4). After multivariate adjustment, TLR ≥ 0.783 was still independently associated with cardiovascular mortality (HR = 2.044, 95%CI 1.264, 3.305, *p* = 0.004, Table 4). The other independent predictor included BMI, NYHA class ≥ 3, and moderate–severe mitral regurgitation (Table 4).

**Table 3:** Univariate and multivariate cox regression analysis of all-cause mortality.

Variables	Univariable		Multivariable	
	HR (95%CI)	*p*	HR (95%CI)	*p*
Age (>65 years old)	2.204 (1.397, 3.478)	0.001	1.795 (1.108, 2.906)	0.017
Male	1.367 (0.926, 2.018)	0.116		
BMI	0.920 (0.876, 0.968)	0.001	0.936 (0.892, 0.983)	0.008
NYHA ≥ 3	2.773 (1.678, 4.583)	<0.001	2.143 (1.284, 3.576)	0.004
Atrial fibrillation	1.092 (0.755, 1.578)	0.640		
NT-proBNP > 450pg./mL	1.621 (0.943, 2.788)	0.081		
LVEF < 50%	1.068 (0.711, 1.603)	0.752		
Moderate–Severe MR	1.386 (0.964, 1.992)	0.078		
Intracellular water	0.952 (0.914, 0.992)	0.018		
Extracellular water	1.047 (0.965, 1.136)	0.273		
Diuretics	0.910 (0.597, 1.385)	0.658		
TLR ≥ 0.783	2.606 (1.773, 3.829)	<0.001	2.108 (1.400, 3.173)	<0.001

BMI, body mass index; NYHA, New York Heart association Functional Classification; NT-proBNP, N-terminal prohormone of brain natriuretic peptide; LVEF, left ventricular ejection fraction; MR, mitral regurgitation; TLR, total body water to lean body mass ratio.

**Table 4:** Univariate and multivariate cox regression analysis of cardiovascular mortality.

Variables	Univariable		Multivariable	
	HR (95%CI)	*p*	HR (95%CI)	*p*
Age (>65 years old)	1.406 (0.869, 2.275)	0.165		
Male	1.550 (0.964, 2.491)	0.070		
BMI	0.900 (0.848, 0.956)	0.001	0.914 (0.860, 0.971)	0.004
NYHA ≥ 3	2.975 (1.613, 5.486)	<0.001	2.137 (1.144, 3.990)	0.017
Atrial fibrillation	1.090 (0.704, 1.688)	0.700		
NT-proBNP > 450pg./ml	1.555 (0.824, 2.933)	0.173		
LVEF < 50%	1.683 (0.976, 2.904)	0.061		
Moderate–Severe MR	2.059 (1.320, 3.212)	0.001	1.774 (1.105, 2.947)	0.018
Intracellular water	0.938 (0.892, 0.986)	0.012		
Extracellular water	0.962 (0.895, 1.034)	0.294		
Diuretics	1.036 (0.616, 1.741)	0.894		
TLR ≥ 0.783	2.417 (1.539, 3.798)	<0.001	2.044 (1.264, 3.305)	0.004

BMI, body mass index; NYHA, New York Heart association Functional Classification; NT-proBNP, N-terminal prohormone of brain natriuretic peptide; LVEF, left ventricular ejection fraction; MR, mitral regurgitation; TLR, total body water to lean body mass ratio.

**Figure 7:**
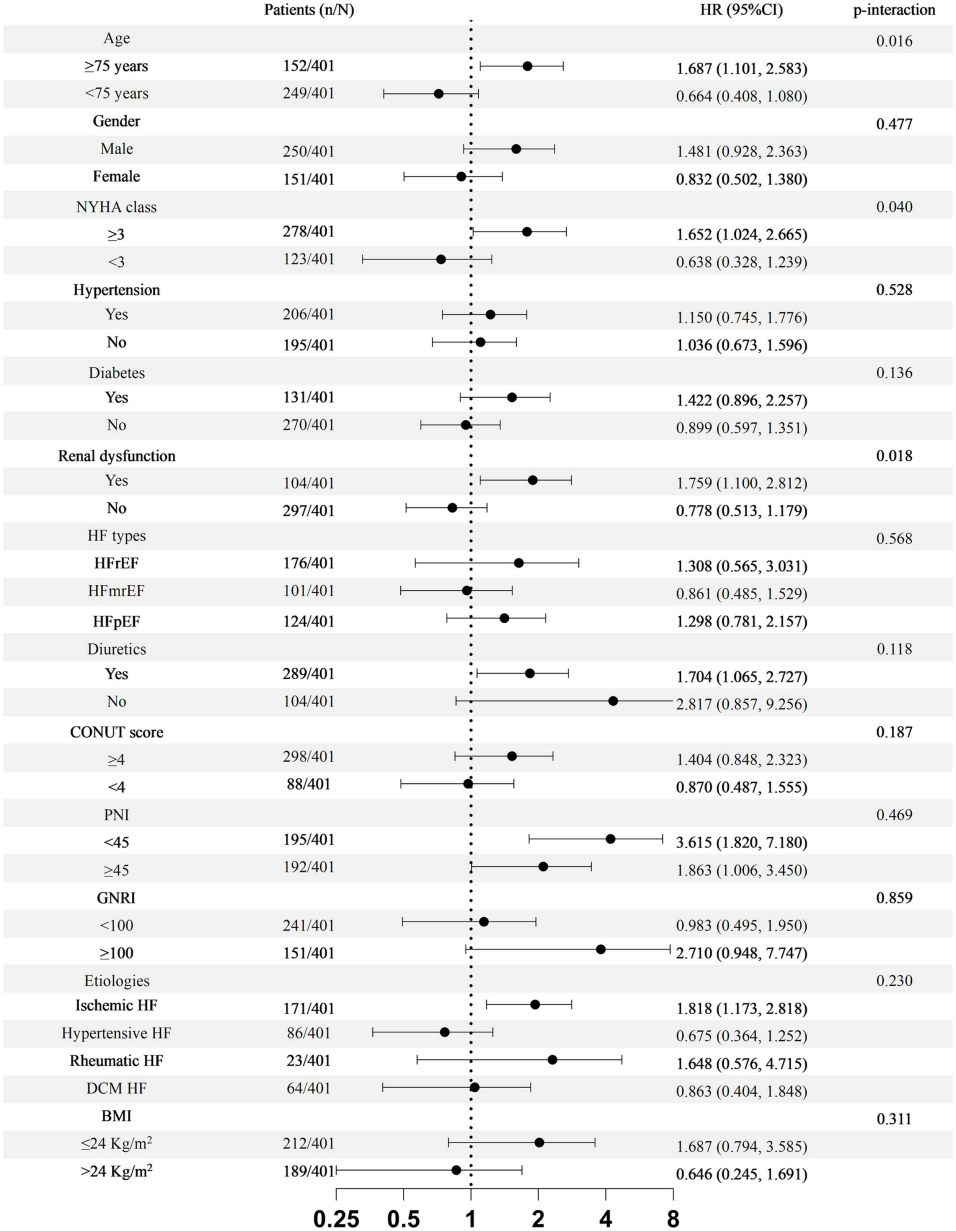
Subgroup analyses for the association of total body water to lean body mass ratio (TLR) with all-cause mortality. HF, heart failure; HFrEF, heart failure with reduced ejection fraction; HFmrEF, heart failure with mildly reduced ejection fraction; HFpEF, heart failure with preserved ejection fraction; COUNT, controlling nutritional status score; PNI, prognostic nutritional index; GNRI, geriatric nutritional risk index; BMI, body mass index.

A more detailed analysis regarding the association between TLR and all-cause mortality was performed and depicted in Figure 7. The interaction-term analysis showed a higher risk of all-cause mortality in association with high-TLR in patients older than 75 years old (*p* = 0.016), NYHA class ≥3 (*p* = 0.040), and history of CKD (*p* = 0.018). Hypertension, diabetes, sex, the use of diuretic, BMI, nutritional status, and types or etiologies of HF did not significantly influence the prognostic value of TLR (all *p* > 0.05).

Sensitivity analyses using tertiles of TLR (low, medium, high) confirmed that patients in the higher tertile had significantly increased all-cause mortality (T2 vs. T1: HR = 2.302, 95%CI 1.311, 4.043, *p* = 0.004; T3 vs. T1: HR = 2.919, 95%CI 1.690, 5.041, *p* < 0.001; Supplementary Table S3) and cardiovascular mortality (T2 vs. T1: HR = 2.544, 95%CI 1.314, 4.924, *p* = 0.006; T3 vs. T1: HR = 2.855, 95%CI 1.492, 5.463, *p* = 0.002; Supplementary Table S4) compared to the lowest tertile, supporting the robustness of the TLR-mortality association across alternative classifications.

## 4 Discussion

The present study mainly demonstrated that: (1) Muscle wasting and fluid retention is one of the most common complications of CHF patients. (2) LBM and TBW itself have a low predictive value for long-term prognosis of CHF, but a ratio between TBW to LBM (TLR) provided a significant improved on predictive power. (3) Increased TLR was associated with a higher risk of long-term all-cause and cardiovascular mortality. (4) After multivariable adjustment, high TLR remained an independent risk factor for long-term all-cause and cardiovascular mortality. (5) TLR showed modest but consistent predictive value for long-term mortality, with AUC and IDI comparable to several established prognostic indicators. Its discriminative ability, while promising, should be interpreted in the context of existing clinical markers. To our knowledge, present study was the first to propose TLR as a novel prognostic biomarker in CHF patients.

The CHF is a major public health that affects about 26 million people worldwide (24). Despite advances in the treatment of HF, malnutrition and cardiac cachexia is still a frequent and neglected condition, which affects between 16 to 62% of patients with the disease (25). To date, there is no clear explanation of how HF is associated with the loss of muscles, fat, and bone mass. Some studies believe that several factors exist to maintain the wasting process, they are activation of neurohormones and proinflammatory cytokines (26), dietary deficiencies (27), gut malabsorption (28), and decreased bowel perfusion (29). Not only that, as the disease progresses, HF patients are often presented with depression, anxiety, and other adverse psychological states that may accelerate the wasting process. CHF patients presented with malnutrition suffer from a worse prognosis, while early diagnosis and treatment were associated with a better prognosis (13, 30), hence, the need for nutritional risk assessment and early intervention is important. Several surrogate markers, such as BMI, PNI, CONUT score, albumin, and lymphocyte counts have been proven to have the ability to reflect the nutritional status and are associated with poor prognosis (31, 32); however, sophisticated algorithms, as well as, inconsistent sensitivity and specificity lead to a demand of more specific biomarker.

The BMI owing to its simplicity is widely used for defining body size and indicating nutritional status, to the extent of assessing the risk of mortality (33). However, BMI’s predictive ability is inconsistent, and also it does not take age, sex, bone structure, muscle mass, and edema into consideration, which may lead to bias and errors in measurement (34–36). Such limitations caused other researchers to focus on different body composition parameters that are more effective for predicting mortality. LBM recently has been proposed as a potential modifiable contributor to the pathophysiology of HF as a result of a positive association between lower LBM with a higher mortality in HF patients (19, 37, 38). In fact, the impact of LBM as a prognostic biomarker is also presented in patients with chronic kidney disease (39) and coronary heart disease (40). Several mechanisms may explain why lower LBM was associated with higher mortality. First, the muscle hypothesis of chronic HF, where a reduction in left ventricular function caused an abnormality in muscular metabolism and function. This leads to excessive sympathetic vasoconstrictor drive and ventilatory response (41). Second, skeletal muscle influences the whole energy metabolism, thus, changes in skeletal muscle will affect energy production and consumption (42). Third, skeletal muscles secrete myokines that may improve insulin sensitivity along with anti-inflammatory and antioxidant effects (43). Fourth, one could hypothesize that higher muscle mass equal to higher fat mass, indicates a better metabolic reserve that may be advantageous in HF, particularly in the setting of stressful situations (44). Our study cohort consisted of CHF patients with a higher risk of malnutrition, unfortunately, the predictive value of LBM was relatively low, possibly due to the differences in population study (37, 38).

In the early stages of CHF, alteration of hemodynamic and neuro-hormonal systems took place as a part of compensatory mechanisms. As the disease worsens, the insufficiency of compensatory adaptive mechanisms to counteract the deleterious effects of decreased blood flow results in further depression of ventricular function and sodium and water retention (45). Congestion is a hallmark feature of CHF leading to cardinal symptoms such as pulmonary congestion and pitting edema (1). This volume overload is not only associated with an increased risk of hospitalization but also with a worse prognosis (20). Chronic volume overload is one of the main reasons for re-hospitalization in patients with acute decompensated HF, and residual congestion at hospital discharge can worsen the outcomes in patients with CHF (46). Of note, achieving complete decongestion in such patients is challenging, making the search for a novel biomarker that may predict the volume status of CHF patients remains valuable. In our previous study, we found that the edema index may reflect one’s congestion status and EI-guided management may be a promising way to achieve euvolemia in patients with CHF (20). However, EI is only able to reflect extracellular volume status, while 70% of LBM is composed of water and may reflect malnutrition status in CHF patients (37, 38). Accordingly, we designed a new biomarker namely TLR, which can reflect one’s volume and nutritional status. In this present study, we found that the prognostic value of TLR was higher than TBW or LBM alone, and increased TLR level was associated with a higher risk of all-cause and cardiovascular mortality.

The exact mechanism of how TLR was associated with poor prognosis in CHF patients remains to be elucidated. Several explanations may unravel such complex mechanisms to some degree. First, a chronic state of inadequate tissue perfusion may lead to a vicious cycle of malnutrition, and inflammation, and eventually lead to cardiac cachexia (47, 48). Cardiac cachexia has an annual mortality of 20–40% with an increase of mortality rate by 50% within 18 months of first diagnosis (7, 8). Secondly, LBM provides an important metabolic benefit. Skeletal muscle not only plays an important role in energy production and consumption systems but is also the main target for insulin-mediated glucose uptake (19). Of note, skeletal muscle as an endocrine organ may secrete myokines, such as interleukin-6 (IL-6), brain-derived neurotrophic factor (BDNF), fibroblast growth factor 21 (FBG-21), decorin (49), and leukemia-inhibitor factor (LIF) (50) that are beneficial for improving cardiac metabolism, increasing insulin sensitivity, promoting anti-inflammatory and antioxidant effects (42, 43). Moreover, previous studies have mentioned that HF may alter the profile of such myokines (51–55) and eventually lead to muscle weakness and wasting. It is noteworthy that muscle wasting is a frequent (19.5%) co-morbidity among patients with CHF and is associated with reduced exercise capacity, muscle strength, and aggravation of HF (56). Thirdly, sodium and water retention is considered an important pathophysiologic mechanism in CHF. Congestion is identical to volume overload, and a combination of TBW and LBM that is composed of 70% water may somewhat reflect the volume status of CHF patients. All in all, we believed that TLR provided greater benefit than other previous biomarkers (5, 21), as it may reflect the nutritional and volume status of CHF patients.

The TBW and LBM can be measured through body composition analyzers, such as dual-energy X-ray absorptiometry (DXA) (57), magnetic resonance imaging (MRI) (58), and BIA (59). DXA is considered the gold standard for body composition assessment and is the most accurate way (60), however, specialized equipment, radiation exposure, and high cost limited its usage in clinical practice (60). Similar to MRI where it may accurately estimate fat and skeletal muscle, such time-consuming and costly examination limited its practical use to analyze body composition (61). In contrast, BIA can be easily used for noninvasive indirect assessment of body composition, and studies have shown that the accuracy of BIA for body composition assessment was non-inferior to DXA (62, 63), indicating the validity of BIA in assessing body composition in daily practice. CHF patients tended to have low physical activity and quality of life, therefore, in this study, we preferred to use BIA as the main body composition analyzer due to its simplicity and convenience for bedside use. To the best of our knowledge, this was the first study to evaluate the prognostic value of TLR in patients with CHF. The present study found that TLR has the ability to reflect one’s volume and nutritional status. After multivariate regression, we found that TLR ≥ 0.783 was an independent prognostic biomarker for all-cause and cardiovascular mortality in CHF patients. This finding indicated that TLR may serve as a novel prognostic biomarker in CHF patients. Last but not least, it is noteworthy that studies regarding the ability of TLR as a prognostic biomarker are relatively scarce and there was no fixed reference value for such a parameter, thus, careful interpretation is needed.

The TLR cut-off of ≥0.783 identified in this study should be interpreted within the context of our single-center cohort and the specific bioimpedance device (InBody S10) used. Body composition measurements, including TBW and LBM, can vary between devices and populations, which may limit the direct transferability of this exact numerical threshold. Therefore, external validation in independent, multi-center cohorts using different BIA devices is necessary to establish a more generalizable cut-off. To assess the robustness of our findings, we performed sensitivity analyses using alternative classifications such as tertiles of TLR, which consistently showed that patients in the highest TLR group had significantly worse outcomes (all *p* < 0.01). Future studies should also explore whether TLR cut-offs differ according to age, sex, or CHF etiology, to further personalize its clinical application.

The AUC of TLR in our study, while modest in absolute terms, compares favorably with that of several established single biomarkers in HF prognosis, such as NT-proBNP and renal function markers, in the same population. This suggests that TLR, as a composite metric derived from a single bedside test, holds practical clinical value. To further enhance its predictive accuracy, future studies could explore: (1) integrating TLR into multi-marker prediction models (e.g., combined with natriuretic peptides or clinical risk scores); (2) calibrating TBW and LBM measurements for specific device models and patient demographics to reduce measurement variability; and (3) assessing the prognostic value of serial TLR measurements over time, which may better capture dynamic changes in fluid and nutritional status than a single baseline value.

The findings of this study suggest that TLR not only serves as a prognostic marker but may also inform personalized nutritional interventions in CHF patients. Unlike traditional biomarkers that reflect either volume status (e.g., NT-proBNP, edema index) or nutritional status (e.g., albumin, CONUT score) alone, TLR integrates both dimensions, offering a more holistic view of the patient’s physiological state. This dual insight can guide clinicians in tailoring dietary strategies according to the predominant derangement. For patients with a high TLR (≥0.783) and signs of volume overload (e.g., edema, elevated jugular pressure), nutritional management should prioritize fluid and sodium restriction, possibly in conjunction with diuretic therapy. However, caution is needed to avoid exacerbating malnutrition; thus, protein and energy intake should be maintained or supplemented as needed. Conversely, in patients with a high TLR accompanied by low muscle mass or biochemical evidence of malnutrition (e.g., low albumin, high CONUT score), the focus should shift toward aggressive protein and energy supplementation—potentially via oral nutritional supplements or enteral nutrition—while closely monitoring fluid balance to prevent worsening congestion. In patients with a low TLR (<0.783), nutritional efforts may be directed at preserving lean mass and preventing future deterioration, with an emphasis on balanced macronutrient intake and regular monitoring for changes in hydration status. TLR could thus serve as a practical tool for dietitians and cardiologists to dynamically adjust nutritional prescriptions in response to changes in body composition and fluid balance over time. Future studies should explore the impact of TLR-guided nutritional interventions on hard clinical endpoints, such as hospitalization rates, functional capacity, and survival. Integrating TLR into multidisciplinary care pathways—alongside established nutritional assessment tools—could enhance the precision of dietary management in CHF and advance the field of personalized nutrition in chronic heart disease.

There are some limitations in our study. First, this was a single-center, prospective observational study with a moderate sample size conducted in a specific regional population. The potential for selection bias and other unmeasured confounders cannot be fully excluded. Consequently, the findings—particularly the derived TLR cut-off value—may not be directly generalizable to populations with different characteristics, such as Western cohorts, obese patients, or those with differing prevalences of heart failure phenotypes (e.g., HFpEF vs. HFrEF), without external validation in multi-center and ethnically diverse cohorts. Second, obese individuals have a relatively high ECW and TBW, thus overestimation of fat-free mass and underestimation of fat-mass may affect the accuracy of BIA (64). Third, while we adjusted for baseline diuretic use in our multivariate models and subgroup analysis, we lacked detailed information on diuretic dosage, duration of therapy, and any changes in diuretic regimens during the follow-up period. As diuretics can rapidly alter TBW, ECW, and ICW, this may have introduced variability in BIA measurements and influenced TLR values. The use of diuretics may also cause an invalid estimation of skeletal muscle (65), fat-free mass, and body fat mass (66). Fourth, although we mentioned that TLR may also reflect the nutritional status of CHF patients, unfortunately, while we assessed nutritional status using CONUT score, PNI, and GNRI, we lacked data from other comprehensive nutritional screening tools such as NRS-2002 and the Mini Nutritional Assessment (MNA). Last but not least, we are aware that right atrial pressure and pulmonary capillary wedge pressure measurement is the golden standard to measure one’s volume status, however, due to high-cost and invasive methods, not all patients underwent right heart catheterization. Finally, the TLR cut-off value was derived from a single-center population using a specific BIA device (InBody S10), and its generalizability to other devices or broader CHF populations requires external validation. Furthermore, while TLR showed promising prognostic value, its discriminative ability (AUC ~ 0.65–0.69) is modest and should not be overinterpreted as superior to established markers such as NT-proBNP or NYHA class in all contexts. Although the present study found that TLR was an independent prognostic biomarker for CHF patients, however, further studies with large sample sizes are still needed to elucidate the connection between TLR with malnutrition and congestion.

## 5 Conclusion

Malnutrition and congestion are some of the most common complications of CHF and are associated with a worse prognosis. Several surrogate markers such as edema index, BNP, BMI, albumin, and nutritional risk stratification scores have been proven to be able to reflect one’s fluid or nutritional status. Unfortunately, to date, no biomarker can reflect both volume and nutritional status. In the present study, we developed a novel composite biomarker, the TBW-to-LBM ratio (TLR), which reflects both volume and nutritional status in CHF patients and shows association with long-term prognosis. While TLR demonstrates promising prognostic value, its clinical utility should be further validated alongside established biomarkers in diverse populations.

## Data Availability

The raw data supporting the conclusions of this article will be made available by the authors, without undue reservation.
