# Supplementary material for: Total body water to lean body mass ratio predicts mortality in patients with chronic heart failure: a prospective, observational study
Source: Front Nutr. 2026 Mar 4;13:1762912. doi: 10.3389/fnut.2026.1762912 (PMC12995761; doi:10.3389/fnut.2026.1762912)
Supplement: Supplementary file 1 [file Table_1.docx]

**Table S1.** A comparison of nutritional status of the 2 groups

| Baseline Characteristics | Total  n= 401 | TLR<0.783  n=212 | TLR≥0.783  n=189 | p value |
| --- | --- | --- | --- | --- |
| CONUT score, M (Q1, Q3) | 5.00 (4.00, 6.00) | 4.00 (3.00, 5.00) | 5.00 (4.00, 6.00) | <.001 |
| PNI, M (Q1, Q3) | 44.85 (41.33, 48.48) | 46.88 (43.63, 50.26) | 42.55 (38.84, 46.47) | <.001 |
| GNRI, M (Q1, Q3) | 98.28 (92.33, 102.75) | 101.26 (95.30, 104.64) | 95.30 (90.40, 99.77) | <.001 |

TLR: total body water to lean body mass ratio; COUNT: controlling nutritional status score; PNI: prognostic nutritional index; GNRI: geriatric nutritional risk index.

**Table S2** The results of integrated discrimination improvement (IDI)

|  | All-cause mortality | | | |
| --- | --- | --- | --- | --- |
| Variables | IDI | | IDI | |
|  | IDI [95% CI] | p | IDI [95% CI] | p |
| TLR vs. HB | 0.053[0.017-0.088] | 0.004 | 0.028[0.000-0.055] | 0.052 |
| TLR vs. Albumin | 0.049[0.020-0.077] | 0.001 | 0.025[0.003-0.047] | 0.024 |
| TLR vs. Creatinine | 0.078[0.049-0.107] | <.001 | 0.037[0.015-0.058] | 0.001 |
| TLR vs. BMI | 0.056[0.024-0.087] | <.001 | 0.01[-0.015-0.035] | 0.439 |
| TLR vs. NT-proBNP | 0.032[-0.005-0.068] | 0.09 | -0.014[-0.05-0.021] | 0.429 |
| TLR vs. CONUT score | 0.039[0.010-0.069] | 0.009 | 0.026[0.004-0.048] | 0.002 |
| TLR vs. GNRI | 0.02[-0.011-0.05] | 0.214 | 0.002[-0.022-0.026] | 0.872 |
| TLR vs. PNI | 0.049[0.02-0.078] | 0.001 | 0.029[0.006-0.052] | 0.014 |
| TLR vs. NYHA class | 0.016[-0.017-0.049] | 0.335 | -0.002[-0.028-0.024] | 0.900 |
| TLR vs. LVEF | 0.065[0.033-0.097] | <.001 | -0.011[-0.044-0.021] | 0.493 |

TLR: total body water to lean body mass ratio; BMI: body mass index; NT-proBNP: N-terminal prohormone of brain natriuretic peptide; COUNT score: controlling nutritional status score; NYHA: New York Heart association Functional Classification; LVEF: left ventricular ejection fraction; CONUT: controlling nutritional status score; PNI: prognostic nutritional index; GNRI: geriatric nutritional risk index.

**Table S3.** Univariate and multivariate cox regression analysis of all-cause mortality

| Variables | Univariable  HR (95%CI) p | | Multivariable  HR (95%CI) p | |
| --- | --- | --- | --- | --- |
| Age (>65 years old) | 2.204 (1.397, 3.478) | 0.001 | 1.838 (1.123, 3.008) | 0.016 |
| Male | 1.367 (0.926, 2.018) | 0.116 | 1.515 (1.007, 2.280) | 0.046 |
| BMI | 0.920 (0.876, 0.968) | 0.001 | 0.948 (0.903, 0.996) | 0.034 |
| NYHA≥3 | 2.773 (1.678, 4.583) | <0.001 | 2.040 (1.221, 3.410) | 0.007 |
| Atrial Fibrillation | 1.092 (0.755, 1.578) | 0.640 |  |  |
| NT-proBNP>450pg/ml | 1.621 (0.943, 2.788) | 0.081 |  |  |
| LVEF<50% | 1.068 (0.711, 1.603) | 0.752 |  |  |
| Moderate-Severe MR | 1.386 (0.964, 1.992) | 0.078 |  |  |
| Intracellular Water | 0.952 (0.914, 0.992) | 0.018 |  |  |
| Extracellular Water | 1.047 (0.965, 1.136) | 0.273 |  |  |
| Diuretics | 0.910 (0.597, 1.385) | 0.658 |  |  |
| T1 of TLR: <0.781 | Reference |  | Reference |  |
| T2 of TLR: 0.781≤TLR<0.785 | 2.377 (1.406, 4.020) | <0.001 | 2.302 (1.311, 4.043) | 0.004 |
| T3 of TLR: ≥0.783 | 3.765 (2.301, 6.161) | <0.001 | 2.919 (1.690, 5.041) | <0.001 |

BMI: body mass index; NYHA: New York Heart association Functional Classification; NT-proBNP: N-terminal prohormone of brain natriuretic peptide; LVEF: left ventricular ejection fraction; MR: mitral regurgitation; TLR: total body water to lean body mass ratio.

**Table S4.** Univariate and multivariate cox regression analysis of cardiovascular mortality

| Variables | Univariable  HR (95%CI) p | | Multivariable  HR (95%CI) p | |
| --- | --- | --- | --- | --- |
| Age (>65 years old) | 1.406 (0.869, 2.275) | 0.165 |  |  |
| Male | 1.550 (0.964, 2.491) | 0.070 |  |  |
| BMI | 0.900 (0.848, 0.956) | 0.001 | 0.919 (0.865, 0.976) | 0.006 |
| NYHA≥3 | 2.975 (1.613, 5.486) | <0.001 | 2.079 (1.113, 3.883) | 0.022 |
| Atrial Fibrillation | 1.090 (0.704, 1.688) | 0.700 |  |  |
| NT-proBNP>450pg/ml | 1.555 (0.824, 2.933) | 0.173 |  |  |
| LVEF<50% | 1.683 (0.976, 2.904) | 0.061 |  |  |
| Moderate-Severe MR | 2.059 (1.320, 3.212) | 0.001 | 1.750 (1.090, 2.811) | 0.021 |
| Intracellular Water | 0.938 (0.892, 0.986) | 0.012 |  |  |
| Extracellular Water | 0.962 (0.895, 1.034) | 0.294 |  |  |
| Diuretics | 1.036 (0.616, 1.741) | 0.894 |  |  |
| T1 of TLR: <0.781 | Reference |  | Reference |  |
| T2 of TLR: 0.781≤TLR<0.785 | 2.403 (1.300, 4.442) | 0.005 | 2.544 (1.314, 4.924) | 0.006 |
| T3 of TLR: ≥0.783 | 3.524 (1.968, 6.311) | <0.001 | 2.855 (1.492, 5.463) | 0.002 |

BMI: body mass index; NYHA: New York Heart association Functional Classification; NT-proBNP: N-terminal prohormone of brain natriuretic peptide; LVEF: left ventricular ejection fraction; MR: mitral regurgitation; TLR: total body water to lean body mass ratio.


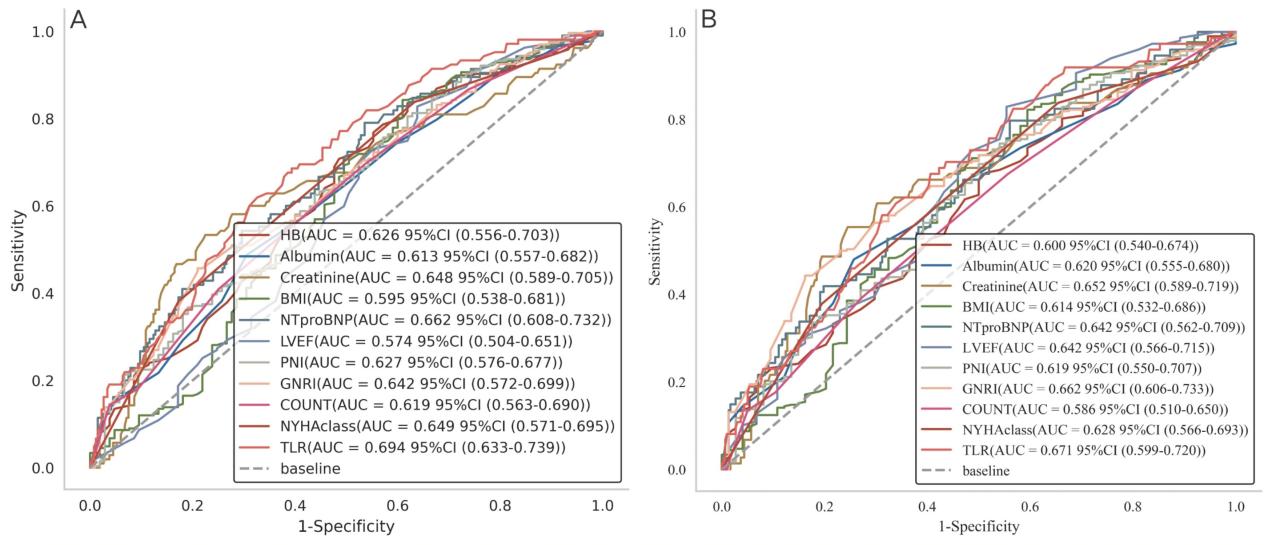


**Figure S1** ROC analysis of total body water to lean body mass ratio (TLR), albumin, creatinine, body mass index (BMI), N-terminal prohormone of brain natriuretic peptide (NT-proBNP), controlling nutritional status score (CONUT score), prognostic nutritional index (PNI), geriatric nutritional risk index (GNRI), left ventricular ejection fraction (LVEF), and NYHA class for predicting long-term all-cause (A) and cardiovascular (B) mortality.
